# Suppression of FGFR3- and MYC-dependent oncogenesis by tubacin: association with HDAC6-dependent and independent activities

**DOI:** 10.18632/oncotarget.22816

**Published:** 2017-12-01

**Authors:** Sara Ota, Zi-Qiang Zhou, Peter J. Hurlin

**Affiliations:** ^1^ Shriners Hospitals for Children Portland, Portland, OR 97239, USA; ^2^ Department of Cell, Developmental and Cancer Biology and Knight Cancer Institute, Oregon Health & Science University, Portland, OR 97239, USA

**Keywords:** fibroblast growth factor receptor (FGFR), histone deacetylase 6 (HDAC6), MYC, cyclin D1, DNA damage

## Abstract

Fibroblast growth factor receptor 3 (FGFR3) is amplified, translocated or mutated in a number of different human cancer types, but most commonly in bladder cancers. We previously found that the accumulation of FGFR3 is dependent on histone deacetylase 6 (HDAC6). Here we show that HDAC6 loss or inhibition reduces FGFR3 accumulation in cells made tumorigenic by ectopic expression of a mutant activated version of FGFR3 together with the MYC oncoprotein and in a bladder cancer cell line whose tumorigenicity is dependent on expression of a translocated version of FGFR3. In tumor xenoplant assays, HDAC6 deficiency or small molecule inhibition by the selective HDAC6 inhibitors tubacin or tubastatin A was found to significantly impede tumor growth. However, tubacin was more effective at inhibiting tumor growth than tubastatin A or HDAC6 deficiency. The superior anti-tumor activity of tubacin was linked to its ability to not only inhibit accumulation of mutant FGFR3, but also to cause robust downregulation of MYC and cyclin D1, and to induce a DNA damage response and apoptosis. Neither HDAC6 deficiency nor treatment with tubastatin A altered MYC or cyclin D1 levels, and neither induced a DNA damage response or apoptosis. Thus while tubacin and tubastatin A inhibit HDAC6 with similar selectivity and potency, our results reveal unique HDAC6-independent activities of tubacin that likely contribute to its potent anti-tumor activity.

## INTRODUCTION

Mutations, translocations and amplification of the FGFR3 gene that lead to increased expression and receptor activity have been found in a variety of tumor types, but most frequently in bladder cancer [1, 2]. Mutations in FGFR3 that increase its expression and activity are found in approximately 10–20% of muscle invasive bladder cancers and 70–80% of low-grade papillary bladder cancers [2, 3]. Approximately 40–50% of muscle invasive bladder tumors overexpress FGFR3 [4, 5]. The involvement of FGFR3 in bladder and other cancers has led to the development of a variety of approaches designed to directly block the oncogenic function of FGFR3. Included in these approaches has been the use of monoclonal antibodies against FGFR3 and a number of small molecule tyrosine kinase inhibitors [6]. In general, these strategies were found to be effective at inhibiting FGFR3 activity, reducing cell proliferation, and in some cases significantly reducing tumor growth in mouse xenoplant assays [5, 7]. However, these strategies were much less effective at inducing apoptosis/cell death, a much more stringent criteria for evaluating the durable effectiveness of any cancer therapeutic. Moreover, human clinical trials using the partially selective FGFR tyrosine kinase inhibitor dovitinib have thus far failed to provide any significant benefit in bladder cancer treatment [8, 9].

In addition to FGFR3, alterations in a number of other genes including *MYC* and *CCND1* (encoding cyclin D1), are found in bladder cancers and thought to contribute to oncogenesis [2, 3, 10–13]. Whereas MYC is a transcription factor that regulates many genes important for cell proliferation, cyclin D1/CDK complexes can phosphorylate and inactivate the retinoblastoma (RB) tumor suppressor protein to propel G1 to S phase progression. Overexpression of MYC and cyclin D1 can cause bypass of cell cycle checkpoints and promote tumor cell proliferation [14, 15]. Cyclin D1 can also be recruited to sites of DNA damage where it participates in the repair of DNA damage [16]. Cyclin D1’s function in facilitating the repair of potentially catastrophic DNA damage is supported by the finding that its depletion can sensitize tumor cells to ionizing radiation-driven cell death [17]. These results suggest that increased cyclin D1 supports oncogenesis by both promoting proliferation and facilitating the repair of increased DNA damage which is typically associated with unbridled proliferation.

We recently found that efficient accumulation of a constitutively active FGFR3 mutant which is responsible for the lethal human disorder thanatophoric dysplasia type II (TDII) and is found in some bladder and other cancer types, was dependent on HDAC6 in cultured cells and *in vivo* [18]. Both small molecule inhibition of HDAC6 and HDAC6 deficiency promoted degradation of mutant FGFR3 and improved skeletal growth in a model of TDII [18]. HDAC6 resides primarily in the cytoplasm and, unlike nuclear HDACs, its major substrates are not histones, but cytoplasmic proteins such as α-tubulin, cortactin and heat shock protein 90 (HSP90) [19, 20]. HDAC6 deficiency and/or inhibition was previously shown to be effective at promoting degradation of epidermal growth factor receptor (EGFR), and related mechanisms, involving altered/accelerated trafficking of FGFR3 and EGFR along microtubules to lysosomes, may be responsible for enhancing their degradation [18, 21–23].

The above findings raised the possibility that HDAC6 inhibition may be an effective therapeutic strategy for FGFR3-dependent cancers. In a previous study, it was shown that HDAC6-selective inhibitors, including tubacin [24] and tubastatin A [25], had anti-proliferative activity and increased apoptosis in urothelial cancer cell lines [26]. Despite these effects on cultured cells, the micromolar drug concentrations needed were considered to be too high to warrant use as an *in vivo* therapeutic and their anti-tumor activities were not tested. However, mice lacking HDAC6 are viable, fertile and generally healthy [27], and *in vivo* studies suggest that even relatively high concentrations of HDAC6 inhibitors are well tolerated [28, 29]. Here, we show that FGFR3-dependent tumors are sensitive to tubacin, tubastatin A and HDAC6 deficiency and reveal unique, HDAC6-independent activities of tubacin that may contribute to its superior ability to block tumor growth.

## RESULTS

### HDAC6 deficiency suppresses the transformed state of cells expressing ectopic FGFR3^K644E^ and MYC

**Figure 1:**
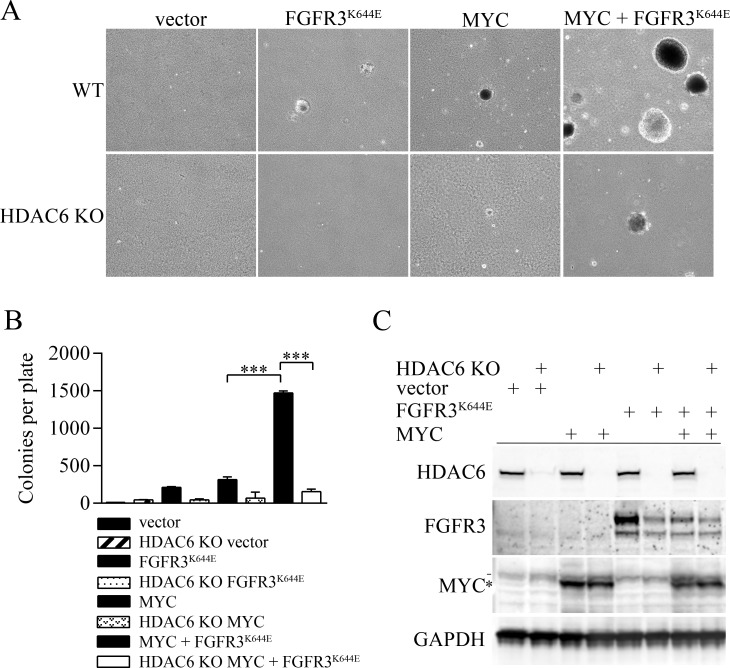
Tubacin and HDAC6 deficiency inhibit soft agar colony formation by cells transformed by FGFR3^K644E^ and MYC (**A**) Representative images of colonies formed in soft agar by wild type MEFs or HDAC6 KO MEF cells expressing empty vector, FGFR3^K644E^, MYC or MYC plus FGFR3^K644E^. (**B**) Mean number of soft agar colonies per plate (*n* = 3) formed by the indicated cell lines. *p*-values were calculated using a one-way ANOVA analysis. ^***^p < 0.0001 (**C**) Immunoblot analysis of the indicated proteins in wildtype immortal MEFs or HDAC6 knockout (KO) MEFs that were infected with empty vector, or with expression vectors for FGFR3^K644E^ or MYC. GAPDH is used as a loading control. ^*^ indicates ectopic MYC and - indicates endogenous MYC.

**Figure 2:**
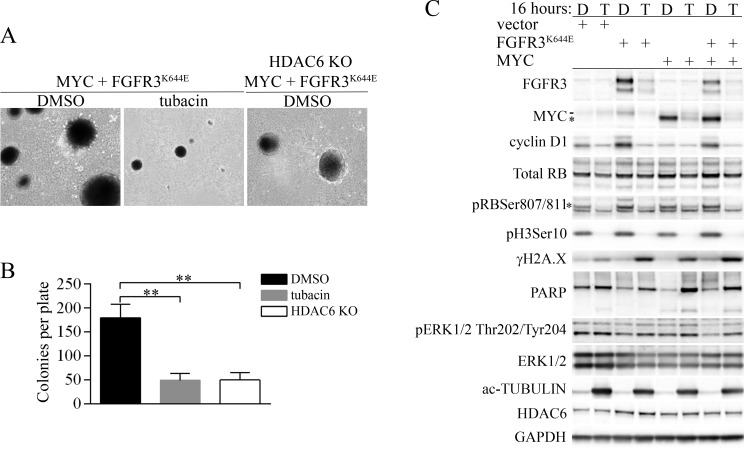
Tubacin inhibits accumulation of FGFR3^K644E^, MYC and cyclin D1 and selectively induces a DNA damage response in MEFs expressing FGFR3^K644E^ and/or MYC (**A**) Representative images of soft agar colonies formed by MEFs expressing FGFR3^K644E^ plus MYC that were treated with either vehicle (DMSO) or 20 µM tubacin, and HDAC6 KO MEFs expressing FGFR3^K644E^ plus MYC that were treated with DMSO. (**B**) The mean number of soft agar colonies per plate is shown (*n* = 3). ^**^p < 0.0005. (**C**) MEFs transfected with empty expression vector (vector) or expressing FGFR3^K644E^, MYC, or both FGFR3^K644E^ and MYC as indicated were treated with DMSO (D) or 20 µM tubacin (T) for 16 hours before cells were collected and lysed. Immunoblots were performed for the indicated proteins. ^*^ ectopic MYC, - endogenous MYC. For pRBSer807/811, the asterisk indicates the primary band specific for pRbSer807/811. GAPDH was used as a loading control.

The K650E/K652E residue in tyrosine kinase domain 2 of FGFR3 (of isoforms IIIB and IIIC respectively) causes constitutive receptor activation and is found in bladder and other cancers [2, 30]. Using colony formation in soft agar as a read-out for cancerous transformation, we found that the ectopic expression of either murine FGFR3^K644E^ (equivalent to human FGFR3^K650E^) or MYC (human c-MYC) in immortalized mouse embryonic fibroblasts (MEFs) led to some cells capable of forming small colonies. When coexpressed, MYC plus FGFR3^K644E^ cooperated to produce cells capable of robust colony formation (Figure 1A, 1B). Expression vectors for FGFR3^K644E^ and MYC alone, or the combination of FGFR3^K644E^ plus MYC, were also introduced into HDAC6 knockout (KO) MEFs [31]. The absence of HDAC6 was associated with a significant reduction in the number of colonies formed by MEFs expressing either FGFR3^K644E^ or MYC alone as well as in the large number of colonies formed by cells expressing both FGFR3^K644E^ and MYC (Figure 1A, 1B). While endogenous FGFR3 was not detectable in vector-transfected control MEFs, the absence of HDAC6 was associated with reduced accumulation of ectopic FGFR3^K644E^ (Figure 1C), a result consistent with our previous study [18]. The absence of HDAC6 also caused a decrease in the mutant receptor in cells expressing both FGFR3^K644E^ and MYC, but basal levels of FGFR3^K644E^ were reduced by ectopic MYC expression itself (Figure 1C). We lack a clear understanding why MYC overexpression causes a reduction in mutant FGFR3, but note that it was reproducibly observed in multiple independent experiments. (e.g. see Figure 2C). The absence of HDAC6 had no effect on either endogenous Myc or ectopically expressed MYC. Overall, these results indicate that soft-agar colony formation by cells transformed with FGFR3^K644E^ and MYC is highly dependent on HDAC6 and that the suppressed colony formation observed is associated reduced accumulation of FGFR3^K644E^.

### HDAC6-independent regulation of FGFR3, MYC, cyclin D1 and DNA damage signaling by tubacin

Similar to the absence of HDAC6, we previously showed that the HDAC6-selective inhibitor tubacin was effective at suppressing the accumulation of mutant FGFR3 [18]. We therefore tested whether tubacin was effective at suppressing soft-agar colony formation by cells transformed by FGFR3^K644E^ plus MYC. As shown in Figure 2A and 2B, 20 µM tubacin was as effective as HDAC6 deficiency in suppressing colony formation, and colonies that did emerge were generally smaller (Figure 1C). Immunoblot analyses carried out 4 hours (Supplementary Figure 1) and 16 hours (Figure 2) after treatment with 20 µM tubacin showed robust downregulation of FGFR3^K644E^ in MEFs expressing ectopic FGFR3^K644E^ alone and in MEFs expressing FGFR3^K644E^ plus MYC (Figure 2).

To further address potential mechanisms that contribute to suppression of the transformed phenotype by tubacin, we examined the expression of MYC and markers of proliferation, DNA damage and apoptosis. Tubacin had little effect on the very low levels of endogenous Myc in vector control cells, but downregulated the increased endogenous Myc found in cells expressing FGFR3^K644E^, and caused rapid and robust downregulation of exogenous MYC in cells transfected by MYC alone or MYC plus FGFR3^K644E^ (Figure 2, Supplementary Figure 1). Similar to Myc, tubacin appeared to have little effect on the low levels of cyclin D1 in vector control cells, but caused rapid downregulation of the high levels of cyclin D1 present in MEFs expressing FGFR3^K644E^ or MYC plus FGFR3^K644E^ (Figure 2, Supplementary Figure 1). The downregulation of cyclin D1 corresponded to reduced abundance of serine 807/811 phosphorylated retinoblastoma protein (pRbSer807/811), a target of cyclin D/cyclin dependent kinase (CDK) complexes [32] and severely reduced abundance of Histone H3 serine 10 phosphorylation (pH3Ser10), an indicator of cells undergoing mitosis [33] (Figure 2, Supplementary Figure 1). Ectopic expression of MYC alone had little or no effect on cyclin D1 levels (Figure 2, Supplementary Figure 1). These results reveal that mutant FGFR3 increases cyclin D1 and MYC expression, and that tubacin is effective at both inhibiting the induction of endogenous cyclin D1 and MYC by the mutant receptor and inhibiting the forced expression of MYC.

Consistent with a previous study [34], each of the cell lines subjected to tubacin induced serine 139 phosphorylation of the variant histone H2A.X (γH2A.X), an early marker for the induction of DNA double-strand breaks and DNA damage signaling [35]. In contrast, PARP cleavage, a marker of apoptosis, was most apparent in tubacin-treated cells expressing ectopic MYC or MYC plus FGFR3^K644E^ treated with tubacin for 16 hours (Figure 2), but not at 4 hours (Supplementary Figure 1). The latter result suggests that elevated MYC, and not FGFR3^K644E^, is responsible for sensitizing tubacin-treated cells to apoptosis.

Finally, all of the cell lines treated with tubacin exhibited strong induction of acetylated α- tubulin as expected, and HDAC6 was modestly increased in cells expressing FGFR3^K644E^ (Figure 2, Supplementary Figure 1). Surprisingly, extracellular signal-regulated kinase (ERK) phosphorylation was not altered by expression of mutant FGFR3 and did not change in response to tubacin (Figure 2, Supplementary Figure 1). These latter results suggest that altered Erk activity does not contribute to the changes in MYC or cyclin D1 abundance in response to mutant FGFR3 or tubacin treatment.

### Mechanisms of cyclin D1 and MYC downregulation by tubacin

**Figure 3:**
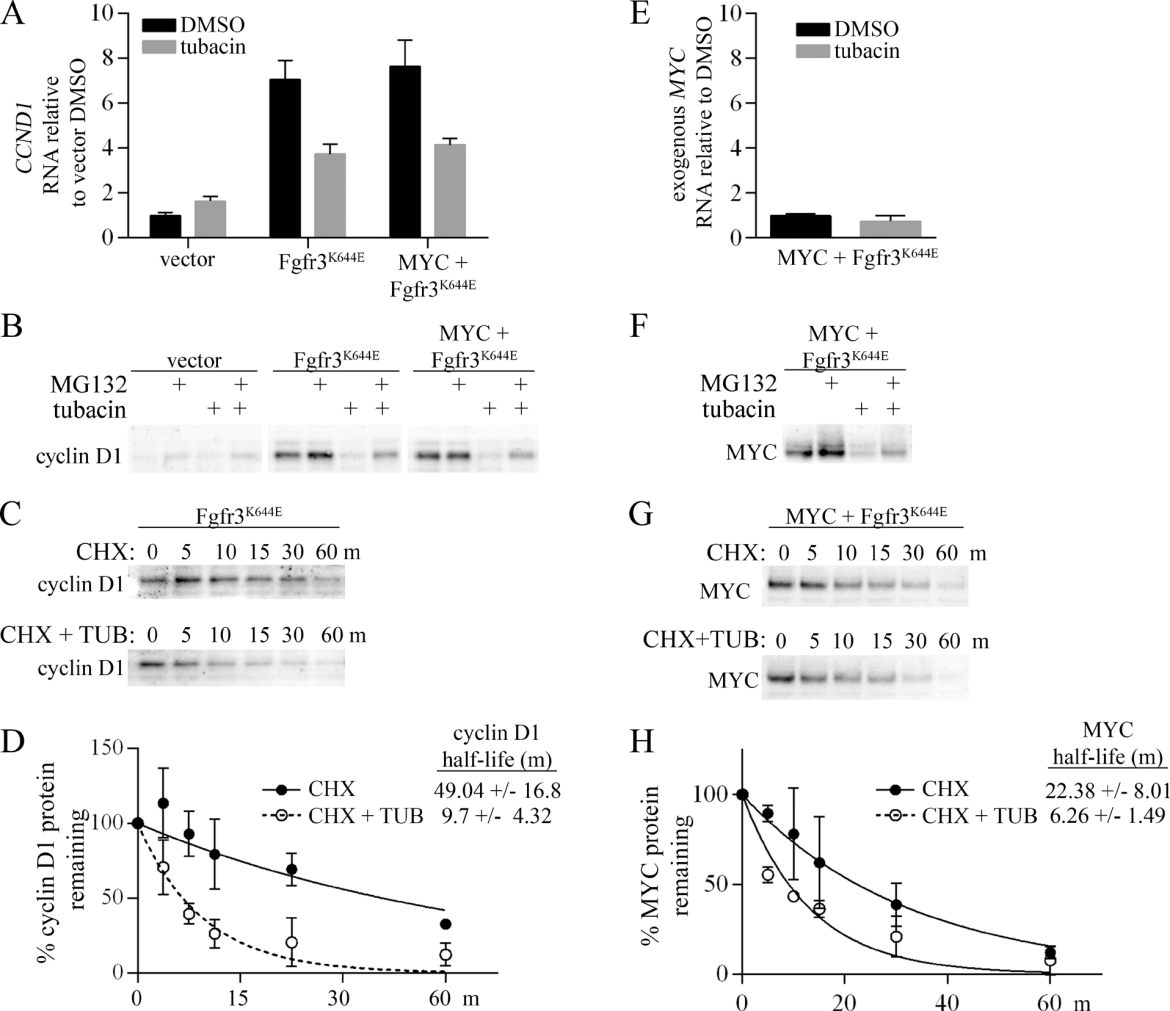
Regulation of cyclin D1 and MYC by tubacin (**A**) Control MEFs infected with empty vectors or expressing ectopic FGFR3^K644E^, or MYC plus FGFR3^K644E^ were treated for 4 hours with either DMSO or 20 µM tubacin as indicated. RNA was harvested in duplicate for qPCR analysis, and qPCR samples were set up in triplicate for analysis (*N* = 6). ΔCT values for *CCND1* expression for each sample were calculated by normalization to *beta-actin*. (**B**) Immunoblots of cyclin D1 following treatment of the indicated cell lines with 10 µM MG132 for 4 hours. (**C**) Representative immunoblot of cyclin D1 from cells incubated with 100 µM cycloheximide (CHX) or 100 µM cycloheximide (CHX) plus 20 µM tubacin (TUB) for the indicated times. (**D**) One-phase decay analysis (*N* = 3) used to determine cyclin D1 half-life. (**E**) Quantitative PCR measuring exogenous MYC in MYC plus FGFR3^K644E^ cells treated with DMSO or 20 µM tubacin for 4 hours (*N* = 6). (**F**) Immunoblots for MYC following treatment of cells with 10 µM MG132 for 4 hours. (**G**) Immunoblots for MYC from MEFs expressing ectopic MYC plus FGFR3^K644E^ incubated with 100 µM cycloheximide (CHX) or 100 µM cycloheximide (CHX) plus 20 µM tubacin (TUB) for the indicated times. (**H**) One-phase decay analysis (*N* = 3) used to determine MYC half-life in tubacin treated and untreated cells.

As shown in Figure 2, cyclin D1 was strongly upregulated in MEFs expressing mutant FGFR3. Consistent with a transcriptional mechanism contributing to the upregulation of cyclin D1, quantitative rtPCR (qPCR) showed an approximately 7-fold increase in *cyclin D1 (CCND1)* RNA in cells expressing mutant FGFR3, with or without ectopic MYC (Figure 3A). Treatment with tubacin caused a significant decrease in *CCND1* RNA within 4 hours (Figure 3A). However *CCND1* RNA levels remained elevated relative to the very low levels of cyclin D1 protein detected 4 or 16 hours after tubacin treatment (Figure 2, Supplementary Figure 1), suggesting both transcriptional and post-transcriptional mechanisms contribute to its downregulation. To determine if tubacin affected cyclin D1 protein stability, we examined cyclin D1 levels following treatment with either the proteasomal inhibitor MG132, or the protein synthesis inhibitor cycloheximide in the presence or absence of tubacin. Treatment of cells with MG132 for 4 hours substantially reversed the tubacin-dependent downregulation of cyclin D1 (Figure 3B). In the presence of cycloheximide, tubacin was found to significantly increase the rate of cyclin D1 degradation (Figure 3C, 3D). Thus, tubacin controls the accumulation of cyclin D1 by both inhibiting the induction of *CCND1* RNA and destabilizing cyclin D1 protein.

In MEFs expressing ectopic MYC plus FGFR3^K644E^, MYC protein was strongly reduced by 4 hours after tubacin treatment (Supplementary Figure 1). As expected, RNA levels of the exogenous *MYC* expressed in these cells was not significantly affected by tubacin treatment (Figure 3E). Similar to cyclin D1, MYC was induced by MG132 and this induction was inhibited by tubacin (Figure 3F). In cycloheximide treated cells, MYC half-life was decreased from approximately 22 minutes to 6 minutes by tubacin (Figure 3G). These results indicate that enhanced proteosomal degradation contributes to the downregulation of MYC caused by tubacin.

### Unique and HDAC6-independent activities of tubacin

**Figure 4:**
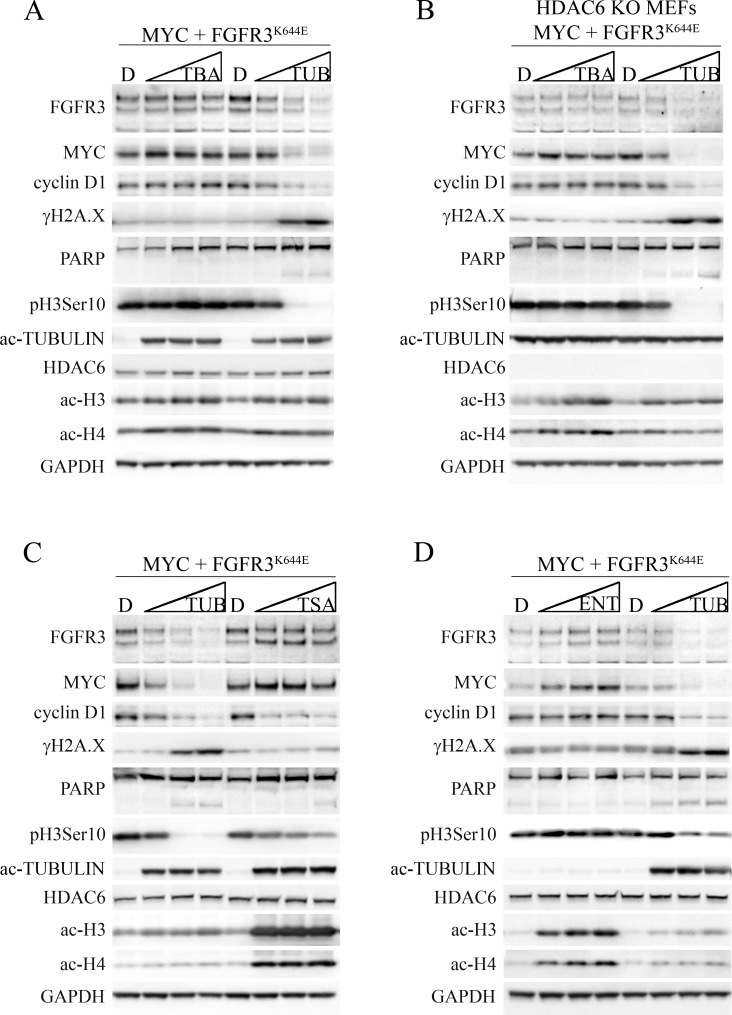
Differential effects of tubastatin A, tubacin, and TSA on FGFR3, MYC, cyclin D1 and DNA damage signaling in MEFs expressing MYC plus FGFR3^K644E^ (**A, C, D**) Immunoblots for the indicated proteins from MEFs expressing MYC plus FGFR3^K644E^ and (**B**) HDAC6 KO MEFs expressing MYC plus FGFR3^K644E^ that were treated with DMSO (D) or 5, 10, or 20 µM tubastatin A (TBA), tubacin (TUB), trichostatin A (TSA) or entinostat (ENT) for 8 hours. GAPDH was used as a loading control.

The ability of tubacin to downregulate MYC and cyclin D1, induce a DNA damage response and suppress FGFR3-dependent cell transformation prompted us to determine whether another HDAC6 inhibitor, tubastatin A, elicited an analogous response. While tubacin is a selective and potent inhibitor of HDAC6 deacetylase activity, tubastatin A has similar potency, but is slightly more selective than tubacin for HDAC6 versus other HDAC family members [25]. MEFs transformed by FGFR3^K644E^ plus MYC were subjected to either tubastatin A or tubacin in a titration format (each drug at 0, 5, 10 and 20 µM) for 8 hours before being harvested for immunoblot analysis. Tubastatin A, like tubacin, induced robust acetylation of α-tubulin and caused downregulation of mutant FGFR3, although tubacin was more effective at decreasing FGFR3^K644E^ (Figure 4A). Unlike tubacin, tubastatin A had no effect on MYC or cyclin D1 abundance and failed to downregulate pH3Ser10 or to induce γH2A.X or PARP cleavage (Figure 4A). The effect of tubacin on mutant FGFR3, MYC, cyclin D1, pH3Ser10 and γH2A.X abundance was dose-dependent, with activity observed at concentrations as low as 5 µM. Both tubacin and tubastatin A only very weakly stimulated acetylation of histones H3 (Lys9/Lys14) and H4 (Lys5/8/12/16) (Figure 4A), consistent with their selective activity towards HDAC6.

To further address the specificity of tubacin and tubastatin A, similar titration experiments were carried out in HDAC6 KO MEFs expressing ectopic MYC plus FGFR3^K644E^. The elevated acetylated α-tubulin found in HDAC6 KO cells was not further increased in HDAC6 KO cells treated with tubacin or tubastatin A, and both drugs caused only very weak induction of H3 and H4 acetylation (Figure 4B). The low level of FGFR3^K644E^ in HDAC6 KO MEFs was not further reduced by tubastatin A treatment (Figure 4B), consistent with the notion that HDAC6 contributes to accumulation of the mutant receptor [18]. As in HDAC6-replete MEFs, tubastatin A had no impact on the abundance of MYC, cyclin D1, pH3Ser10, γH2A.X or PARP cleavage (Figure 4B). In contrast, tubacin caused a further, dose-dependent decrease in FGFR3^K644E^ and its strong regulatory impact on MYC, cyclin D1, pH3Ser10, γH2A.X and PARP cleavage was completely independent of HDAC6 (Figure 4B). Finally, in contrast to wild type and HDAC6 KO MEFs expressing FGFR3^K644E^ plus MYC, neither vector-infected wildtype nor HDAC6 KO MEFs showed PARP cleavage when subjected to tubacin despite similar impacts on MYC, cyclin D1, pH3Ser10 and γH2A.X (Supplementary Figure 2). These latter data further suggest that the transformed state caused by combined FGFR3^K644E^ plus MYC expression imparts selective sensitivity to tubacin-induced apoptosis that is HDAC6 independent.

Although we saw weak and comparable induction of H3 and H4 acetylation by tubastatin A and tubacin (Figure 4A, 4B), the finding that tubacin is more broadly active than tubastatin A against HDACs [25] raised the possibility that its unique activities described in Figure 4A may be due to it targeting other HDACs. To address this, we compared the activities of tubacin with the pan-HDAC inhibitor trichostatin A (TSA) and entinostat (MS-275), which selectively targets the class 1 HDACs, HDAC1 and HDAC3 and does not inhibit HDAC6 [36]. TSA induced robust acetylation of histones H3 and H4 and α-tubulin as expected (Figure 4C). However, unlike tubacin, TSA had little or no effect on the abundance of FGFR3^K644E^ or MYC, and was far less effective than tubacin at inhibiting pH3Ser10, inducing γH2A.X and causing PARP cleavage (Figure 4C). TSA and tubacin did show comparable activity in downregulating cyclin D1 (Figure 4C). The downregulation of cyclin D1 by TSA is consistent with previous studies showing that that TSA both inhibits cyclin D1 transcription [37] and promotes cyclin D1 protein degradation [38]. Entinostat induced acetylation of histone H3 and H4, but not α-tubulin, and had little or no effect on MYC, cyclin D1, pH3Ser10, γH2A.X or PARP cleavage (Figure 4D). Thus while the activities of tubacin that affect FGFR3 accumulation appear to be at least partly dependent on the presence of HDAC6, its activities responsible for regulation of MYC, cyclin D1 and proliferation appear to be entirely HDAC6-independent and independent of inhibition of HDAC1 and HDAC3. Further, the activities of tubacin responsible for provoking DNA damage signaling and PARP cleavage are also largely, if not wholly due to unique and HDAC-independent activities of tubacin.

### Tubacin is effective at suppressing tumor formation by MEFs expressing FGFR3^K644E^ and MYC

**Figure 5:**
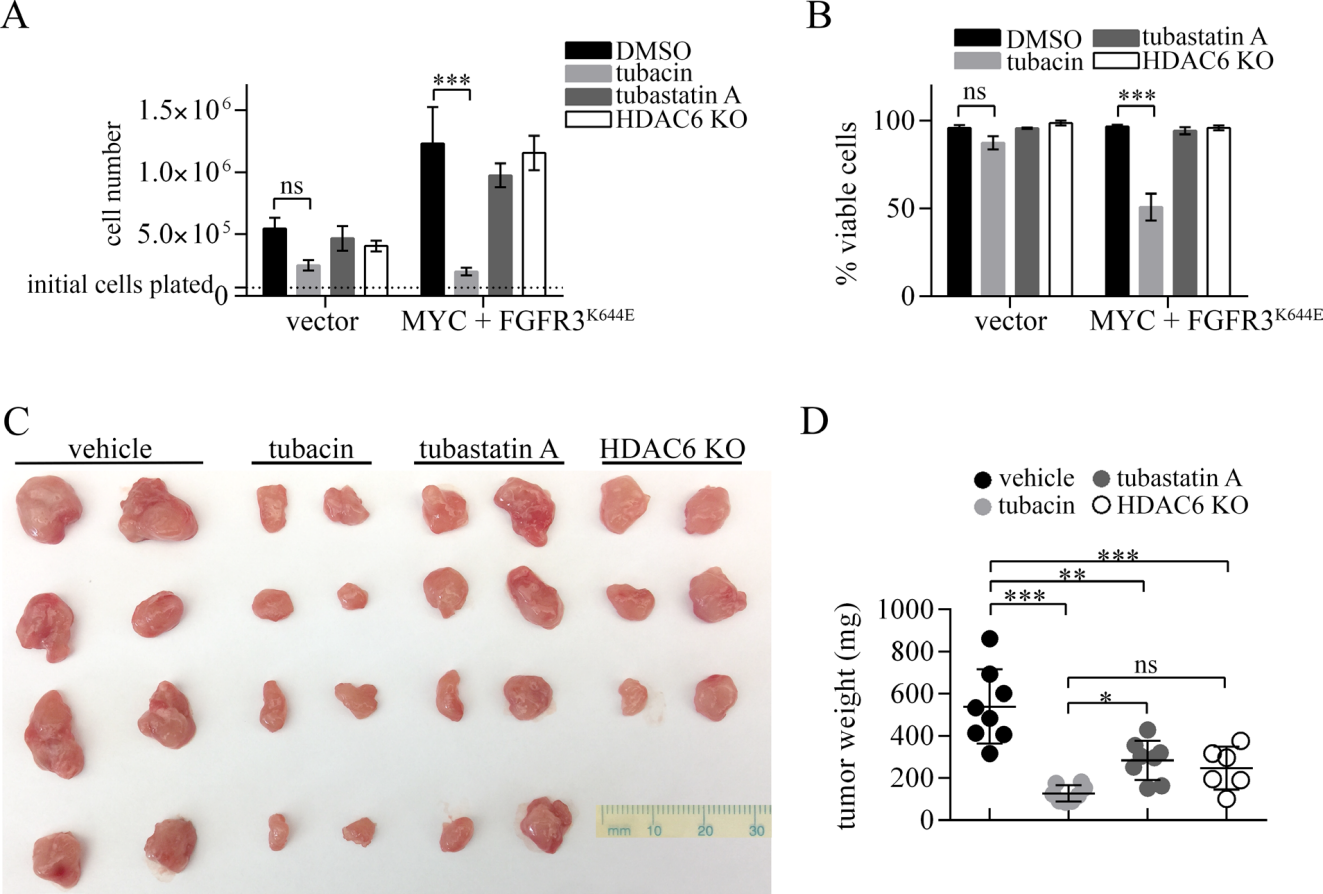
Comparative effects of tubacin, tubastatin A and HDAC6 deficiency on proliferation, viability and tumor formation by MEFs expressing MYC plus FGFR3^K644E^ (**A, B**) 70,000 control MEFs (vector) or MEFs expressing MYC plus FGFR3^K644E^ were plated one day prior to treatment with DMSO, 20 µM tubastatin A, or 20 µM tubacin. (A) Cell numbers and (B) percent of viable cells based on trypan blue exclusion were determined 24 hours after the addition of drug (*N* = 3). ns = non-significant, ^***^p < 0.0001. (**C**) Photographs of excised tumors (**D**) Dot plot analysis of tumor weights of tumors formed by MEFs expressing MYC plus FGFR3^K644E^ in mice treated with vehicle, tubacin or tubastatin A, and tumors formed by HDAC6 KO MEFs expressing MYC plus FGFR3^K644E^. ns, not significant; ^*^p < 0.02; ^**^p = 0.0001, ^***^p < 0.0001.

The activities of tubacin described above suggested that it may be effective in suppressing FGFR3-dependent tumor formation, and that it would be more effective than tubastatin A or HDAC6 deficiency. To test this, we first compared the effects of tubacin, tubastatin A (both at 20 µM) and HDAC6 deficiency on cell proliferation and cell viability using control (vector infected) MEFs and MEFs expressing FGFR3^K644E^ plus MYC. Tubacin strongly blocked proliferation of cells expressing FGFR3^K644E^ plus MYC, and caused a modest decrease in the proliferation of control cells after 24 hours (Figure 5A). Similarly, tubacin caused an approximately 50% decrease in viability of MEFs expressing FGFR3^K644E^ plus MYC, but resulted in only a small and insignificant decrease in the number of viable control cells (Figure 5B). In contrast to tubacin, tubastatin A or HDAC6 deficiency only weakly inhibited cell proliferation and viability of control and FGFR3^K644E^ plus MYC expressing MEFs (Figure 5A, 5B).

The ability of MEFs expressing ectopic FGFR3^K644E^ plus MYC to form tumors was tested in xenoplant assays using Foxn1^nu/nu^ mice. These cells were strongly oncogenic, with subcutaneous injection of 5 × 10^5^ cells leading to the formation of visible tumor nodules by 3 days, which rapidly grew into tumors approximately 2 cm in diameter by 15 days. (Figure 5C, 5D). To compare the potential tumor inhibitory activity of tubacin and tubastatin A in this setting, mice were treated with either tubacin or tubastatin A every other day beginning 3 days post cell injections when tumor nodules became apparent until day 15, when all tumors were excised, weighed and photographed (Figure 5C, 5D). Tubacin strongly inhibited tumor formation, with tumors from treated mice weighing approximately ten times less than in control mice (Figure 5D). Both tumors from tubastatin A treated mice and tumors formed by HDAC6 deficient MEFS were significantly smaller than control tumors, but were not as small as those in tubacin-treated mice (Figure 5C, 5D). These results suggest that reducing the level of mutant FGFR3, a common outcome of both tubacin and tubastatin A treatment and loss of HDAC6, may contribute to the tumor suppression observed, but that the additional activities of tubacin with respect to inhibiting MYC and cyclin D1 and inducing DNA damage signaling may contribute to its superior ability to kill tumor cells and suppress tumor growth.

### Tumor suppressive activities of tubacin in bladder cancer cells

**Figure 6:**
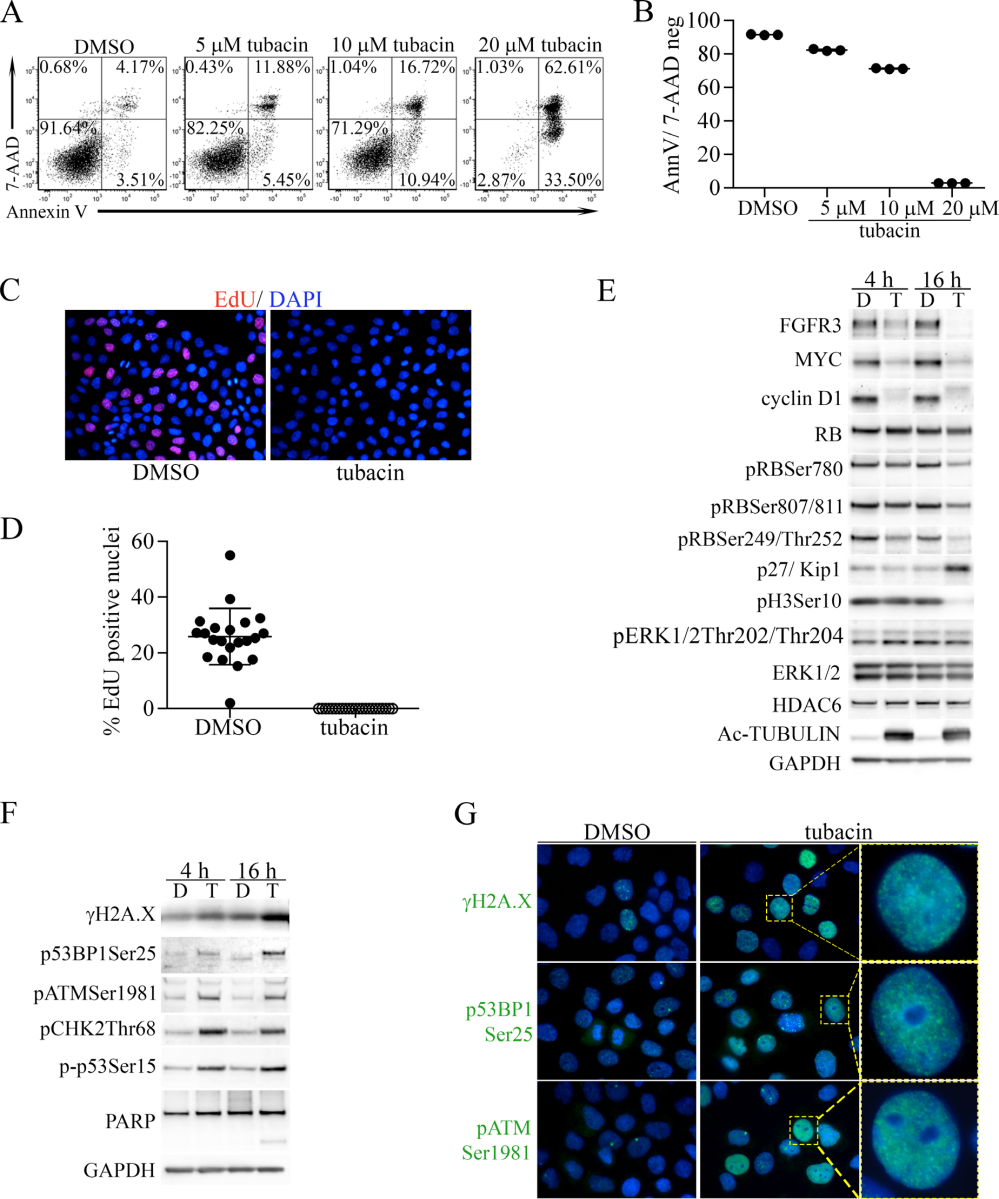
Induction of apoptosis and proliferative arrest by tubacin in RT112 bladder cancer cells is associated with downregulation of FGFR3, MYC, cyclin D1 and induction of DNA damage (**A**) Representative FACS analysis of 7-AAD- and Annexin V-stained RT112 cells treated with DMSO vehicle or the indicated concentrations of tubacin for 48 hours. (**B**) Dot plot of results from three experiments examining apoptosis at the indicated tubacin concentrations. (**C**) Representative image of staining for EdU (red) incorporation in DNA by RT112 cells treated with DMSO or tubacin for 16 hours. Cells were co-stained with DAPI (blue) to mark nuclei. (**D**) Dot plot analysis showing the percentage of EdU positive nuclei per image (N) (DMSO, *N* = 21; tubacin, *N* = 23). (**E**) Immunoblots for FGFR3 (FGFR3-TACC) and the indicated proteins and phospho-proteins associated with cell proliferation and cyclin D1 activity in RT112 cells treated with DMSO (D) or 20 µM tubacin (T) for 4 or 16 hours. (**F**) Immunoblots for the indicated proteins associated with DNA damage signaling, DNA repair and apoptosis from RT112 cells treated with DMSO (D) or 20 µM tubacin (T) for 4 or 16 hours. (**G**) Immunofluorescent staining of the indicated proteins (green) in RT112 cells treated for 16 hours with DMSO or 20 µM tubacin. DAPI stained nuclei are blue. Higher magnification images of the indicated cells (yellow boxes) showing punctate staining patterns are shown at right.

The bladder cancer-derived cell line RT112 expresses high levels of a FGFR3-TACC3 fusion protein [39] and is highly dependent on FGFR3 for its malignant phenotype [7, 40]. RT112 cells were previously found to be moderately sensitive to apoptosis caused by tubacin at 10 µM [26]. Based on the unique activities of tubacin discovered in the MEF model system, we reevaluated the effect of 0, 5 and 10 µM tubacin on apoptosis of RT112 cells and also included 20 µM tubacin. 48 hours after treatment Annexin V (early apoptosis) and 7-AAD (late apoptosis) stained cells were analysed by FACs. Consistent with previous results [26], both 5 and 10 µM tubacin caused some cell death, but a threshold effect was observed with the jump to 20 µM as nearly all cells were killed at this dose (Figure 6A, 6B). We also examined the effect of 20 µM tubacin on cell proliferation 16 hours after treatment. Treated RT112 cells failed to incorporate the UTP analog 5-Ethynyl-2′-deoxyuridine (EdU) into DNA (Figure 6C, 6D), indicating that a severe block in cell proliferation preceded cell death. Immunoblot analyses 4 and 16 hours after treatment showed that the tubacin-induced proliferation block corresponded to rapid and robust downregulation of FGFR3-TACC, MYC, cyclin D1 and pH3Ser10 (Figure 6E). Some PARP cleavage was seen at 16 hours, but not at 4 hours post treatment (Figure 6E). The downregulation of cyclin D1 was associated with decreased phosphorylation of pRB and with increased expression of the cyclin-dependent kinase inhibitor 1B (p27/Kip1) (Figure 6E). Unlike cyclin D1, cyclins D2, E1, E2, A and B1 were only slightly reduced or not downregulated at all (Supplementary Figure 3), and despite the decrease in FGFR3-TACC, there was no change in the abundance of phosphorylated ERK (Figure 6E).

The proliferation block and subsequent cell death caused by 20 µM tubacin also corresponded to induction of DNA damage signaling as indicated by increased γH2A.X as well as DNA-damage-associated phosphorylation of 53BP1, ATM, CHK2 and p53 (Figure 6F). Immunofluorescence staining demonstrated induction of γH2A.X, p53BP1Ser25 and pATMSer1981in a punctate pattern consistent with localization at sites of DNA-damage (Figure 6G).

In contrast to downregulation of FGFR3-TACC fusion protein by 20 µM tubacin, tubastatin A had little or no effect on FGFR3-TACC abundance and TSA had the opposite effect and increased FGFR3-TACC abundance 8 hours post treatment (Supplementary Figure 4). There appears to be a threshold effect for tubacin-induced downregulation of FGFR3-TACC in RT112 cells as doses less than 20 µM failed to cause any decrease. As in MEFs, Tubastatin A failed to cause downregulation of MYC, cyclin D1 and pH3Ser10, and did not induce a DNA damage response in RT112 cells (Supplementary Figure 4). TSA, like tubacin, was effective at downregulating MYC and cyclin D1, but tubacin was far more effective at inducing γH2A.X and DNA damage signaling (Supplementary Figure 4). Entinostat, like TSA induced robust acetylation of Histones H3 and H4 and increased FGFR3-TACC, but unlike TSA did not cause any downregulation of MYC, cyclin D1 or pH3Ser10, and did not induce γH2A.X or other indications of the DNA damage response (Supplementary Figure 4). There was no increase in PARP cleavage with any of the treatments at the 8 hour time, but at least for tubacin, PARP cleavage was apparent by 16 hours (Figure 6F). Thus the unique activity profile of tubacin in RT112 cells, like in the MEF system, suggested that it may be particularly well-suited as a therapeutic for FGFR3-dependent human cancers.

**Figure 7:**
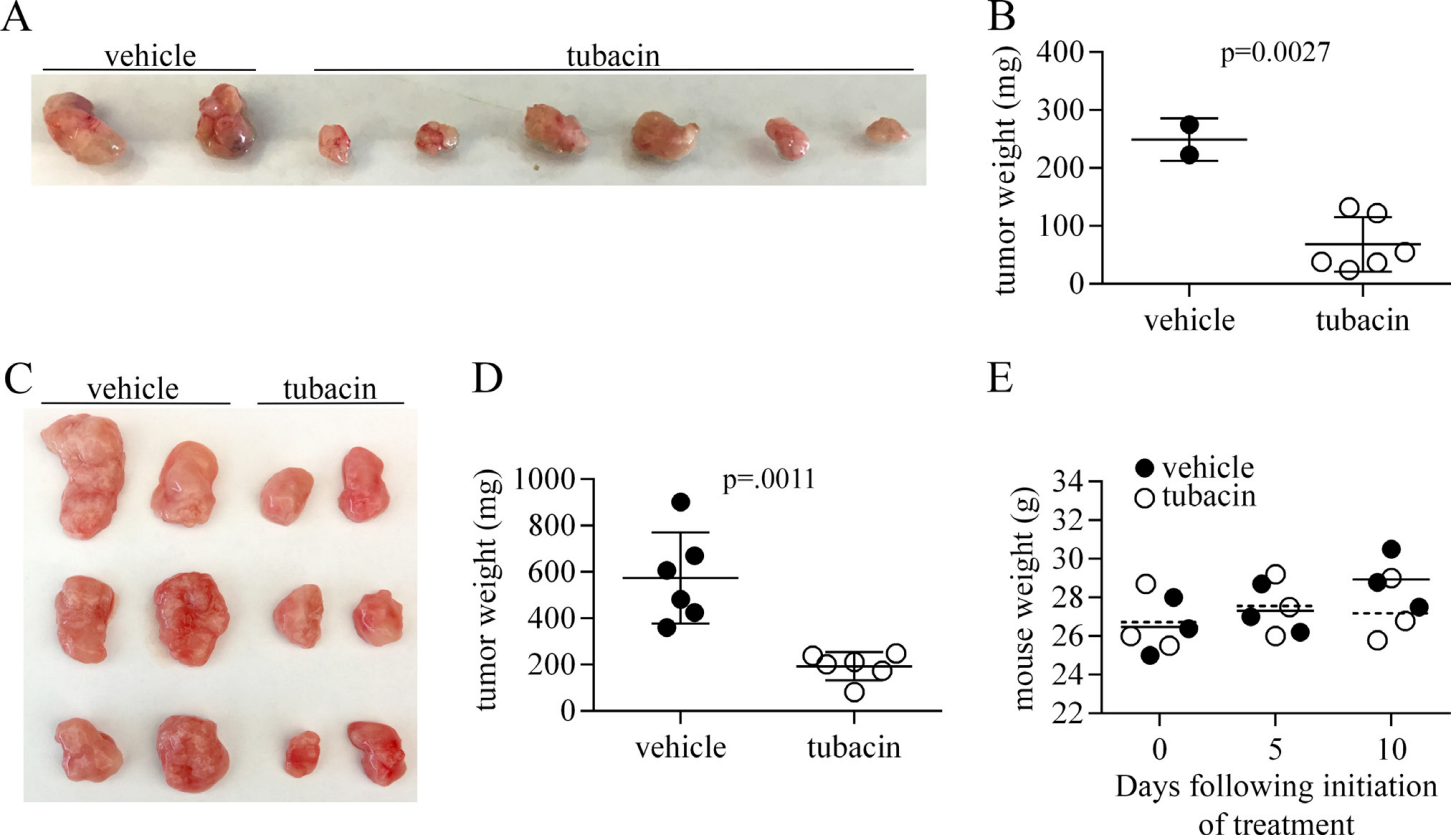
Tubacin inhibits tumor growth by RT112 bladder cancer-derived cells (**A**) Tumors excised from mice that were generated following injection of 2 × 10^6^ RT112 cells and either treated with vehicle or tubacin beginning on day 14 when tumor nodules became visible. (**B**) Dot plot showing weight of tumors in (A). (**C**) Tumors excised from mice that were generated following injection of 5 × 10^6^ cells and either treated with vehicle or tubacin beginning on day 12 when established tumors were approximately 0.5 cm diameter. (**D**) Dot plot showing weight of tumors in (C). (**E**) Dot plot of the weight of vehicle- and tubacin-treated mice (*N* = 3) used in experiments associated with (C). Stippled line – mean weight of tubacin-treated mice, solid line – mean weight of vehicle-treated mice.

To test the efficacy of tubacin at inhibiting the growth of tumors formed by RT112 cells, two experimental protocols were used. In the first, 2 × 10^6^ cells were injected subcutaneously in the shoulder flank of Foxn1^nu/nu^ mice, and treatment of these mice with 25 mg /kg tubacin commenced 14 days later when tumor nodules first became visible. Tubacin IP injections were performed every other day until day 40, at which time mice were euthanized and tumors excised, imaged and weighed (Figure 7A, 7B). In the second protocol, 5 × 10^6^ cells were injected and treatment with 25 mg /kg tubacin commenced 12 days later when tumors were fully established (∼0.5 cm). Tubacin injections were performed every other day until day 22, when the control tumors reached approximately 2 cm in diameter and the mice were euthanized. The excised tumors and their weights are shown in Figure 7C, 7D. In this second set of experiments mice were weighed before treatment, 5 days after treatment began, and after the last treatment. Treatment with tubacin had no significant effect on mouse weight and the small increased weight of control mice relative to tubacin-treated mice at the end of the protocol can largely be accounted for by the increased tumor weight (Figure 7E). No other indications of distress were observed in tubacin-treated mice. These data show that tubacin significantly inhibits RT112 tumor growth and suggest that it is well-tolerated.

## DISCUSSION

The finding that HDAC6 deficiency or inhibition suppressed the accumulation of both wild type FGFR3 and mutant activated FGFR3 [18] led to studies performed here that tested the effectiveness of tubacin and HDAC6 inhibition as a therapeutic strategy in FGFR3-dependent cancers. The engineered model used for these studies was developed by coexpressing the constitutively active FGFR3^K644E^ mutant plus MYC and provided a platform for comparing the effects of HDAC6 inhibitors in oncogenically transformed cells and their non-transformed parental counterparts. Interestingly, in this MEF system, expression of mutant FGFR3 was found to strongly upregulate cyclin D1. The *CCND1* gene is amplified in a variety of cancers, including approximately 20% of muscle-invasive and non-muscle-invasive bladder cancers [2], and its upregulation by mutant FGFR3 is therefore predicted to contribute to FGFR3-dependent oncogenesis. Expression of mutant FGFR3 in MEFs also caused a moderate increase in basal MYC levels, and MYC’s well-described oncogenic activities may be important in FGFR3-dependent cancers. Despite upregulation of cyclin D1 and MYC, ectopic expression of mutant FGFR3 alone only weakly transformed MEFs but cooperated with ectopic MYC expression in causing robust oncogenic transformation.

Tubacin and tubastatin A were previously shown to have similar selectivity and potency as HDAC6 inhibitors, but we found that they exerted markedly different activities in the MEF model system employed. While tubastatin A, tubacin and HDAC6 deficiency all caused robust hyperacetylation of α-tubulin and downregulated expression of mutant FGFR3, tubacin was the most effective at downregulating FGFR3. Tubacin was unique in that it also caused robust downregulation of cyclin D1 and MYC and, as previously described for prostate cancer-derived cells [34], induced DNA damage signaling. Essentially the same contrasting response between tubacin and tubastatin A observed in the MEF system was seen in RT112 bladder cancer-derived cells.

The previously reported ability of tubacin to induce DNA damage signaling in prostate cancer cells was considered to be HDAC6-dependent [34]. However, our experiments performed in HDAC6 KO MEFs demonstrated that induction of the DNA damage response was largely, if not wholly, independent of HDAC6, as was the ability of tubacin to inhibit accumulation of MYC and cyclin D1. These unique and HDAC6-independent activities of tubacin were associated with its ability to selectively inhibit the proliferation and induce apoptosis of cells transformed by FGFR3^K644E^ plus MYC. In contrast, tubastatin A or HDAC6 deficiency had little or no effect on the proliferation or viability of FGFR3^K644E^ plus MYC transformed MEFs, and were less effective than tubacin at suppressing tumor growth by these cells.

Despite their inability to impact the proliferation or viability of FGFR3^K644E^ plus MYC transformed MEFs, both tubastatin A and HDAC6 deficiency did significantly reduce tumor growth by these cells. HDAC6 was previously shown to be required for efficient oncogenic transformation by mutant activated Ras and the absence of HDAC6 slowed the development of DMBA-induced skin tumors [31]. Together, these results suggest that increased signaling in the Ras pathway, as well as other potentially oncogenic pathways [41] operating independently or in some cases together with mutant FGFR3 or other proteins regulated by HDAC6 such as EGFR [21–23], may contribute to reduced tumor growth caused by HDAC6 deficiency or inhibition. The reduced tumor growth in these settings, as well as the reduced ability of HDAC6 deficient MEFs to form colonies in soft agar, suggests that activities specific to HDAC6, perhaps related to cell-cell adhesion, clonegenic growth or the ability of cells to aggregate in the absence of preferred substratum structures, play important roles in tumor formation [41–43]. By inhibiting HDAC6, tubacin is predicted to engage both these HDAC6-dependent anti-tumor activities and the additional HDAC6-independent activities of tubacin identified here. This unique activity profile of tubacin is predicted to contribute to its anti-proliferative and pro-apoptotic effects and make it a superior anti-cancer agent compared to compounds such as tubastatin A that are more HDAC6-specific.

Our results indicate that the tubacin-induced, HDAC6-independent downregulation of both MYC and cyclin D1 occurred at least in part through enhanced proteosomal degradation. Although MYC protein stability can be modulated by its ERK dependent phosphorylation, this does not appear to be responsible for the decreased MYC expression observed since neither expression of mutant FGFR3 nor tubacin-induced downregulation of FGFR3^K644E^ altered the activation status of ERK. Additionally, the finding that the pan-HDAC inhibitor TSA failed to downregulate MYC in MYC-overexpressing MEFs suggests that the relatively weak inhibitory activity of tubacin on HDACs other than HDAC6 is likely not involved in causing MYC downregulation in these cells. In contrast to its lack of effect on MYC, TSA was effective at suppressing accumulation of cyclin D1 in FGFR3^K644E^ plus MYC transformed MEFs, suggesting that tubacin may promote cyclin D1 protein degradation through a mechanism related to inhibition of an HDAC(s) other than HDAC6 or through some unknown non-specific target(s) of tubacin. HDAC1 and HDAC3 can be ruled out since the HDAC1- and HDAC3-selective inhibitor entinostat was not effective in either MEFs or RT112 cells at causing downregulation of cyclin D1. It is also possible that HDAC6-independent (and HDAC1- and HDAC3-independent) induction of DNA damage signaling by tubacin may engage pathways that contribute to the loss of cyclin D1, as well as MYC. Indeed, cyclin D1 has been shown to be downregulated at both the transcriptional and protein stability levels in response to DNA damage [44, 45]. The downregulation of cyclin D1 may also contribute to apoptosis in this setting since cyclin D1 also plays an important role in DNA repair [17] and when left unrepaired the accumulated DNA damage can trigger apoptosis and cell death. DNA damage signaling can also downregulate MYC through the induction of p53 and p53-dependent up regulation of miR-145, which in turn blocks MYC expression through both transcriptional and post-transcriptional mechanisms [46], but not by affecting MYC stability. Nonetheless, it may be interesting to determine whether the DNA damage signaling caused by tubacin results in the induction of miR-145 and contributes to the downregulation of MYC observed in MEFs and RT112 cells. It will also be important to define how tubacin induces DNA damage and the interplay between DNA damage signaling and expression of MYC and cyclin D1.

The unique activity profile of tubacin identified here suggests that it may be effective as a therapeutic agent in not only FGFR3-dependent cancers, but other cancers in which cyclin D1 and/or MYC dysregulation are drivers. In addition to bladder cancer, FGFR3 translocations and amplification of the *CCND1* and *MYC* genes are common in multiple myeloma [47–49]. Although the role in oncogenesis of FGFR3 fusion proteins caused by translocations in multiple myeloma has not been entirely resolved [2], it is interesting that these tumors have been associated with increased cyclin D1 [47, 48], and that HDAC inhibitors have been found to have some therapeutic benefit in multiple myeloma. One such inhibitor is ricolinostat, which was partially derived from the chemical structure of tubacin and has a similar activity profile with respect to HDAC inhibition [50]. However, ricolinostat did not cause downregulation of cyclin D1 in lymphoma cell lines [51], and it remains to be determined whether ricolinostat or other HDAC6 selective inhibitors being developed [52, 53] are effective at downregulating MYC and FGFR3 or other FGFRs. Thus while the relatively poor solubility and high doses of tubacin needed for *in vitro* and *in vivo* responses have limited enthusiasm for advancing tubacin as a therapeutic for humans, the tumor suppressive activities described here suggest that tubacin, or drugs related to tubacin that retain both its HDAC6-dependent and HDAC6-independent activities, may be potent anti-cancer agents.

## MATERIALS AND METHODS

### Cell lines and plasmids

MEF cell lines established from wild type or HDAC6 KO MEFs (a gift from Dr. Tso-Pang Yang) were cultured in high glucose DMEM (Invitrogen). RT112 cells (provided by Dr. William Horton) were cultured in RPMI (Invitrogen). 10% FBS (Hyclone) and 100 U/ml Penicillin and 100 µg/ml Streptomycin (Invitrogen) were included in all cell culture media. Stable MEF cell lines were made by sequential infection using retrovirus produced in EcoPack2 293 cells (Clontech) and selection with geneticin and/or puromycin. Vectors used were control pFBneo and pBabepuro, pFBneoFGFR3^K644E^ (mus), and pBabepuro- MYC (hum).

### Chemicals and antibodies

Chemicals: Crystal Violet (Sigma), cycloheximde (Sigma), DMSO (Sigma), EdU (Invitrogen), entinostat (Selleckchem), Geneticin (Invitrogen), MG132 (Cayman), Noble Agar (Difco), puromycin (Sigma), trichostatin A (Cayman), tubastatin A (Cayman), tubacin (Selleckchem). Primary Antibodies: pH3Ser10 ab47297 (Abcam), p21/Kip1 610241 RB 554136 (BD), p53BP1ser25 A300-652A (Bethyl), cyclin D1 CC12 (Calbiochem), ac- α-Tubulin #5335, GAPDH #2118, HDAC6 #7612, PARP #9542, Ph-p53Ser15 #9284, pHistone H2A.X Ser139 #2577, pChk2Thr68 #2197, pERK1/2 Thr402/Tyr404 #4370, pRbSer780 #9307, pRbSer807/811#9308 (Cell Signaling Technology), FGFR3 sc-123, c-MYC sc-764, cyclin A sc-751, cyclin B1 sc-752, cyclin D2 sc-754, cyclin E sc-481, cyclin E2 sc-28351, ERK1 sc-94 (Santa Cruz Biotechnology), pATMSer1981 #05-740, ac-Histone H3 #06-599, ac-Histone H4 #06-866 (Upstate).

### Proliferation, viability and apoptosis assays

For MEF cell proliferation and viability assays, 70,000 cells were plated per well into 12-well dishes in triplicate for each condition tested. Drug treatment was started 1 day after plating, and simultaneous assessment of proliferation and viability was done using the Invitrogen Countess to count live (trypan blue negative) and dead (trypan blue positive) cells 24 hours after addition of inhibitor. For RT112 cell apoptosis assays, cells were plated in triplicate at a density of 1 × 10^5^ cells per well of a 24-well plate and following drug treatment for 48 hours, cells were trypsinized, washed, and stained with Annexin V-FITC and 7-AAD for analysis using a Becton Dickenson LSRII instrument. Analysis was done using FCS Express 4 (De Novo).

### Immunoblotting

Cells were lysed with RIPA buffer (50 mM Tris–HCl pH 8.0, 150 mM NaCl, 0.1% SDS, 1.0% NP-40, 0.5% sodium deoxycholate) containing COMPLETE Protease Inhibitor cocktail (Roche). Protein quantification was performed using the Bio-Rad DC Protein Quantitation assay. Equal amounts of total protein were prepared in Novex LDS Sample Buffer + 100 µM DTT and run on 1 mm thick, 26-well, 4–12% Novex Bis-Tris midi gels in MOPs running buffer. Semi-Dry Blotting with Novex 2x Transfer buffer was used to transfer protein onto Millipore Immobilon P (PVDF) membranes. Sigma-Aldrich anti-rabbit IgG-Alkaline Phosphatase, anti-mouse-IgG Alkaline Phosphatase, and Anti-rat IgG Alkaline Phosphatase secondary antibodies were used with Amersham ECF Substrate for detection using a BioRad Chemi-Doc MP.

### Quantitative rtPCR

MEF cell lines were plated in duplicate and treated with DMSO or 20 µM tubacin for 4 hours. Total RNA was purified from cellular lysates following the RNeasy Mini Kit protocol (Qiagen) using QIAshredder columns for homogenization. Equal amounts of RNA were used for cDNA syntheses performed using New England Biolabs ProtoScript II First Strand cDNA Synthesis Kit with primer d(T)_23_ VN. RT-PCR reactions were performed in triplicate using a Bio-Rad iQ5 rt-PCR machine with iTaq Universal SYBR Green Supermix. Relative quantitation was calculated based on the ΔCT reference gene method. *Beta-Actin* was used as the reference gene. Primers: *Beta-Actin*: Forward 5′-AGGTCATCACTATTGGCAACGAGC-3′. Reverse 5′-GCACTGTGTTGGCATAGAGGTCTTTA-3′. *CCND1*: Forward 5′-GAGAACAAGCAGACCATCCGC-3′. Reverse 5′ gcaggagaggaagttgttggg-3′. Exogenous human *MYC*: Forward 5′ ATGAGGAGACACCGCCCAC-3′. Reverse 5′ gctgtgaggaggtttgctgtg-3′.

### Protein degradation and proteasomal inhibition assays

Protein half-life experiments using cycloheximide (100 µg/ml) and protein accumulation assays using MG132 (10 µM) were performed as previously described [18]. Cells were co-treated with 20 µM tubacin where noted. Protein accumulation and half-lives were determined from at least three independent experiments. Specific protein quantitation was performed by densitometry using Image J software, and data points were plotted on a non-linear scale with time 0 being set at 100%. The protein half-life was calculated using a one-phase decay equation (GraphPad Prism version 6.07, GraphPad Software, La Jolla California USA, www.graphpad.com).

### Edu incorporation and immunocytochemistry

For analysis of EdU incorporation, cells were grown on tissue culture grade coverglasses and incubated with 10 µM EdU for the final 30 minutes before harvest. Cells were washed with PBS, fixed with 4% paraformaldehyde for five minutes at room temperature then washed again with PBS prior to permeabilization with 0.3% triton-X 100 in PBS. EdU staining was performed using the Click-iT Plus EdU Imaging kit (Molecular Probes). For immunocytochemistry, cells were treated as above and blocked with either 20% donkey or goat serum for 30 minutes. Primary antibody was diluted in permeabilization buffer, applied to cells, and incubated overnight at 4°C. Cells were washed three times with PBS then stained with secondary antibodies for 45 minutes at room temperature. Cy3-conjugated Donkey Anti-Mouse F(ab)2 (Jackson ImmunoResearch Laboratories) or Goat anti-Rabbit IgG (H+L) Superclonal Alexa Fluor 488 (Invitrogen) were used as secondary antibodies. Cells were washed three times with PBS prior to mounting on slides with Permafluor aqueous mounting medium (Thermo Scientific) for imaging. Cells were stained with DAPI during the second to last PBS wash.

### Soft agar colony formation assays

Cells were suspended into 0.4% noble agar in complete media (DMEM + 10% FBS + 1% Pen/Strep) and 1.5 ml was overlaid onto a bottom layer (3 ml) of solidified 0.8% agar in complete media. Cells were fed with complete media every 3 days. For experiments where tubacin was included, 20 µM tubacin was added to top agar when cells were plated and at each feeding (after removal of old medium) until the experiment was terminated at 14 days. Colony formation was determined from plates stained with 0.2% crystal violet in 95% ethanol and scanned using an Epson V550 Photo Scanner. The images were analyzed using Cell Profiler software (www.cellprofiler.org) to identify and count colonies.

### Tumor xenoplant assays

For MEF cell lines, 5 × 10^5^ cells were suspended in PBS and injected subcutaneously (sc) into the shoulder flank of nude mice (Foxn1^nu/nu^, Taconic). For RT112 cells either 2 × 10^6^ or 5 × 10^6^ cells were injected. Drug treatments commenced either when tumor nodules first became apparent (after three days for MEF experiments and 14 days after injection of 2 × 10^6^ RT112 cells) or when tumors formed by injection of 5 × 10^6^ RT112 cells had reached approximately 0.5 cm diameter (12 days). Drug or vehicle injections were conducted every other day until day 40 for mice injected with 2 × 10^6^ cells or for mice injected with 5 × 10^6^ cells when tumors in vehicle treated mice (control tumors) reached approximately 2 cm in diameter–the maximum allowable size under IACUC regulations. The day after the last treatment, mice were sacrificed and the tumors excised, weighed and photographed. For drug treatment, 1 mg/ml tubacin (25 mg/kg) or 1 mg/ml tubastatin A (25 mg/kg) was administered via intraperitoneal injection. Tubacin was dissolved in DMSO and diluted into 5% DMSO, 10% cremophor EL and 85% 0.1 M sodium bicarbonate, pH 9.0. Tubastatin A was dissolved in PBS, pH 7.

## SUPPLEMENTARY MATERIALS FIGURES



## Abbreviations

7-AAD: 7-aminoactinomycin D
53BP1: tumor suppressor p53-binding protein
P53: cellular tumor antigen p53
ATM: ataxia telangiectasia mutated kinase
CHK2: checkpoint kinase 2
EGFR: epidermal growth factor receptor
FGFR3: Fibroblast growth factor receptor 3
GAPDH: Glyceraldehyde-3-phosphate dehydrogenase
HDAC: histone deacetylase
γH2A.X: phosphorylated Histone H2A.X Ser139
H3: Histone H3
pH3Ser10: Phosphorylated Histone H3 Serine 10
H4: Histone H4
IACUC: Institutional Animal Care and Use Committee
MYC: myelocytomatosis viral oncogene
PARP: Poly [ADP-ribose] polymerase
Rb: retinoblastoma-associated protein
RIPA: radioimmunoprecipitation assay buffer
