# Supplementary material for: Suppression of FGFR3- and MYC-dependent oncogenesis by tubacin: association with HDAC6-dependent and independent activities
Source: Oncotarget. 2017 Dec 1;9(3):3172–87. doi: 10.18632/oncotarget.22816 (PMC5790455; doi:10.18632/oncotarget.22816)
Supplement: Supplementary file 1 [file oncotarget-09-3172-s001.pdf]

Suppression of FGFR3- and MYC-dependent oncogenesis by tubacin; association with HDAC6-dependent and independent activities

SUPPLEMENTARY MATERIALS

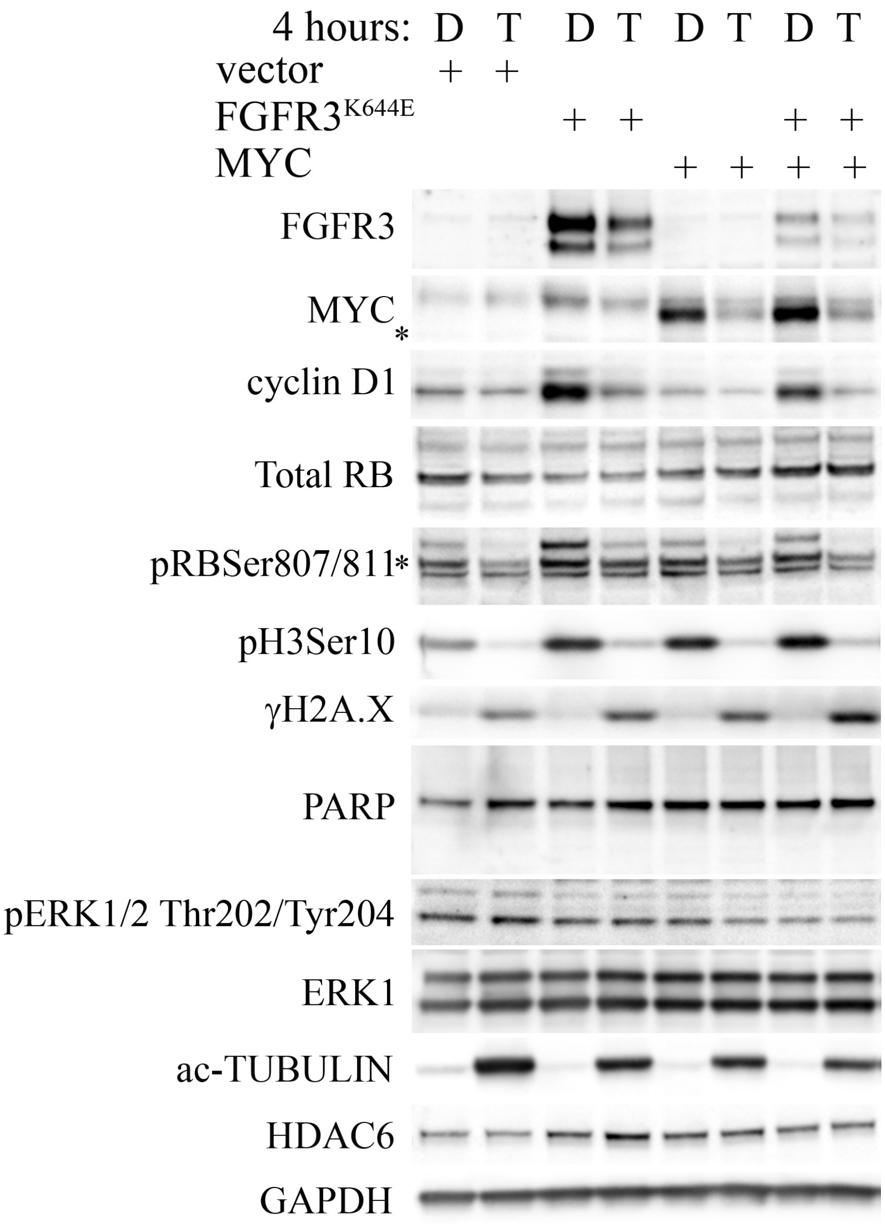

Supplementary Figure 1: MEFs transfected with empty expression vectors (vector) or expressing Fgfr3^{K644E}, MYC, or both Fgfr3^{K644E} and MYC as indicated were treated with DMSO (D) or 20 µM tubacin (T) for 4 hours before cells were collected and lysed. Immunoblots were performed for the indicated proteins. *ectopic MYC, - endogenous Myc. For pRbSer807/811, the asterisk indicates the primary band specific for pRbSer807/811. Gapdh was used as a loading control.

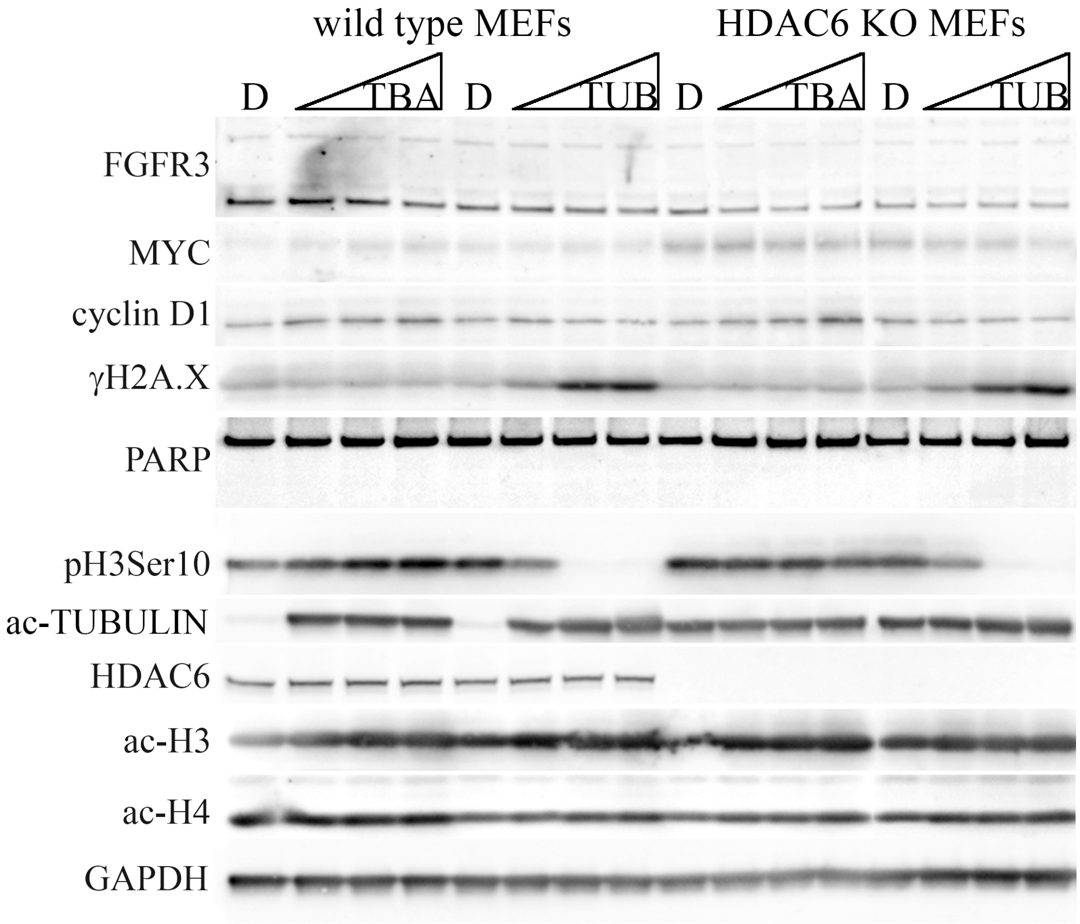

Supplementary Figure 2: The indicated MEFs were treated with 0, 5, 10, or 20 μM tubastatin A (TBA) or tubacin (TUB) for 8 hours, and immunoblots were performed for the indicated proteins. Gapdh was used as a loading control.

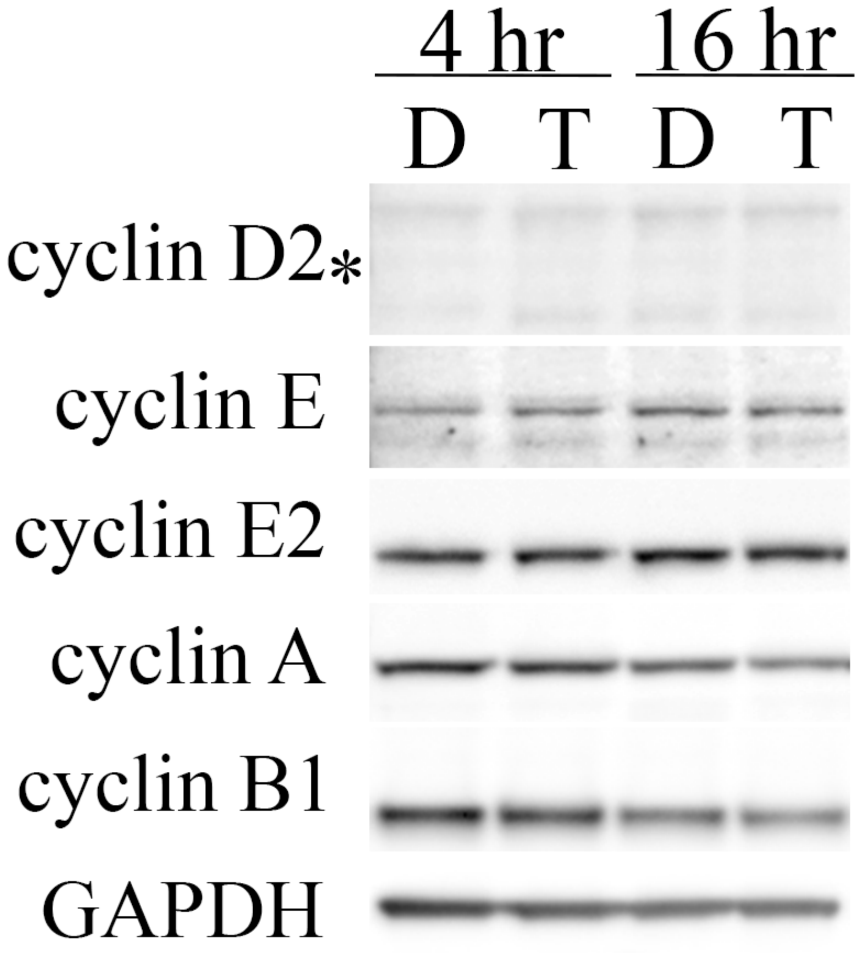

Supplementary Figure 3: Immunoblots for the indicated cyclin proteins from RT112 cells treated with DMSO (D) or 20 µM tubacin (T) for 4 or 16 hours as indicated. *approximate size of cyclin D2 protein, which was essentially non-detectable in RT112 cells. GAPDH was used as a loading control.

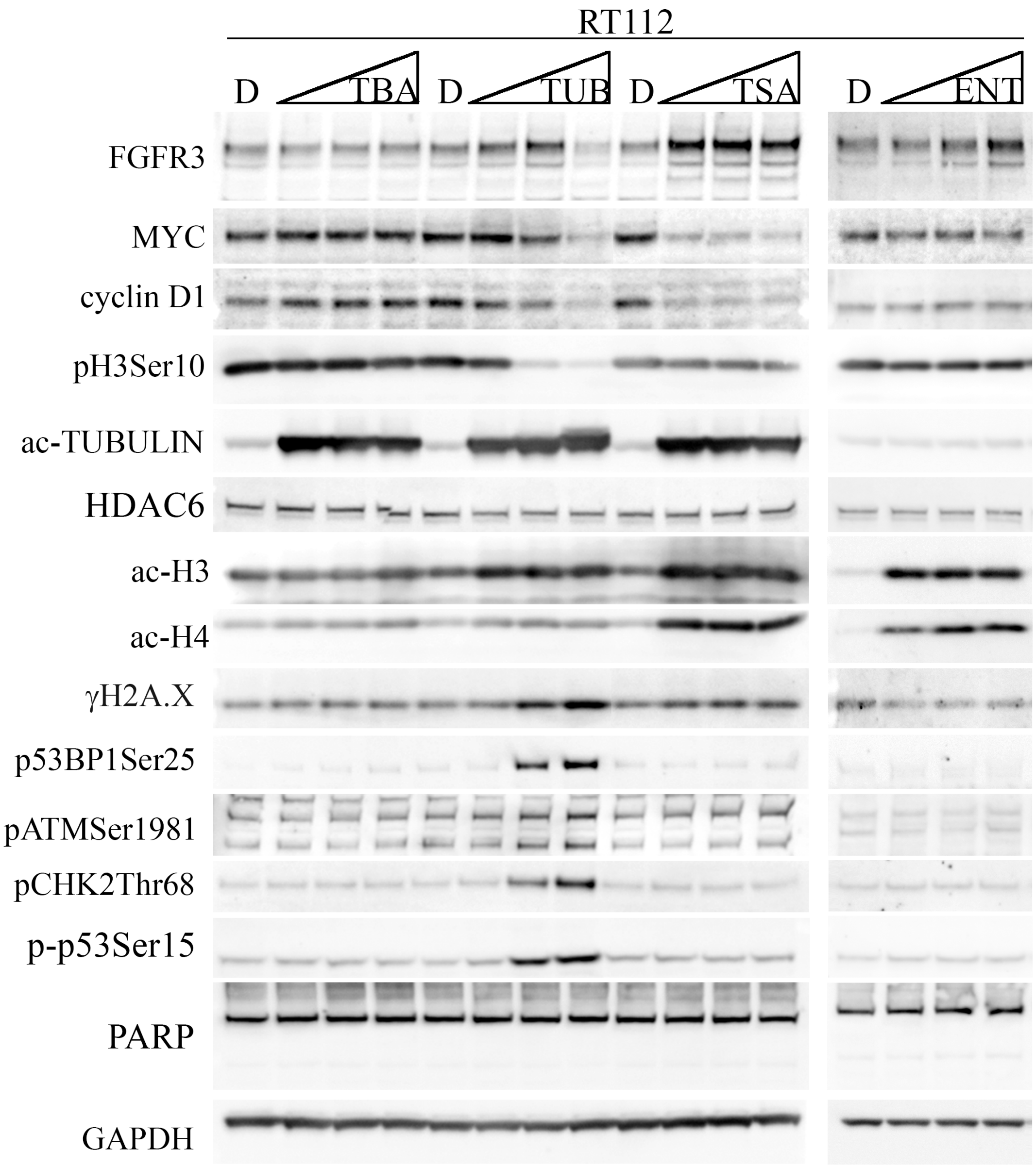

Supplementary Figure 4: Immunoblots for the FGFR3 (FGFR3-TACC) and the indicated proteins and phospho-proteins associated with cell proliferation, DNA-damage signaling and apoptosis from RT112 cells treated with DMSO (D) or 5, 10, or 20 µM tubastatin A (TBA), tubacin (TUB), trichostatin A (TSA) or entinostat (ENT) for 8 hours. Ac-TUBULIN is used as a control for inhibition of HDAC6 activity and ac-H3 and ac-H4 are used as controls for broader HDAC inhibition. GAPDH was used as a loading control.
